# Specific genes of the dopaminergic (*dop-3*) and serotonergic (*tph-1*) pathways contribute to the effects of ethanol consumption in *Caenorhabditis elegans*

**DOI:** 10.1371/journal.pone.0344966

**Published:** 2026-03-23

**Authors:** Teresa Rubio-Tomás, Cynthia A. Hunn, Gábor Hajdú, Csaba Sőti, Nektarios Tavernarakis, Csaba Barta

**Affiliations:** 1 Institute of Molecular Biology and Biotechnology, Foundation for Research and Technology, Heraklion, Crete, Greece; 2 Department of Molecular Biology, Institute of Biochemistry and Molecular Biology, Semmelweis University, Budapest, Hungary; 3 Department of Basic Sciences, Medical School, University of Crete, Heraklion, Crete, Greece; BSRC Alexander Fleming: Biomedical Sciences Research Center Alexander Fleming, GREECE

## Abstract

**Background:**

Excessive alcohol consumption is a global health issue and a leading cause of disease, disability, and mortality. This study aimed to determine the effects of a 24-hour ethanol exposure, post-exposure withdrawal (cessation of alcohol intake), and post-exposure withdrawal relief on the sensorimotor performance of the nematode *Caenorhabditis elegans*.

**Methods:**

A modified kinetic chemotaxis assay (commonly referred as “diacetyl race”) was conducted with worm populations subjected to three different doses of ethanol pre-exposure to assess the impact of ethanol on locomotion. Additionally, we employed lifespan, mobility, gene expression analysis and imaging assays to evaluate health status and molecular alterations occurring in the worms under different levels of ethanol exposure.

**Results:**

Wild-type, dopamine receptor mutant and serotonin biosynthesis null mutant worms presented different responses to ethanol in the kinetic chemotaxis assay. Furthermore, exposure to ethanol altered vesicle exocytosis in dopaminergic and serotonergic neurons and the expression of a panel of genes associated with stress responses. Additionally, 24-hour ethanol exposure differentially influenced the lifespan of wild-type and mutant worms.

**Conclusions:**

Different responses, which may be relevant to the pathogenesis of human alcohol use disorder, were observed in wild-type worms, a dopamine receptor mutant, and a serotonin biosynthesis null mutant in a variety of assays performed. Furthermore, we present a 3-step experimental model for drug tolerance, based on the well-established kinetic chemotaxis behavioral paradigm (“diacetyl race”). This model provides new insights into the effects of alcohol in worms, particularly regarding the roles of dopamine and serotonin neurotransmission. Importantly, this model holds potential for investigating the effects of other addictive substances beyond alcohol.

## Introduction

Excessive alcohol consumption is a significant global health challenge, contributing to disease burden and premature mortality [1]. Despite widespread awareness of its risks, the precise mechanisms through which alcohol induces addictive behaviors are not completely understood. A plethora of model organisms have been employed in the field of alcoholism, with *Caenorhabditis elegans* (*C. elegans*) emerging as a particularly insightful model due to its unique biological characteristics and suitability for high-resolution genetic studies [2–5].

*C. elegans* is a small, transparent nematode that has become one the main models used in neurobiological research owing to its simple nervous system as well as genetic manipulation [6,7]. This nematode offers a concise yet comprehensive platform for investigating complex neuropathological processes due to its fully mapped nervous system, consisting of 302 neurons and over 7,000 chemical synapses and gap junction connections in the hermaphroditic worm [8]. The relevance of *C. elegans* to addiction research is underpinned by its physiological and behavioral responses to addictive substances such as ethanol. Studies have shown that ethanol exposure affects the locomotion, feeding, egg-laying and hatching behaviors of the worm in a dose-dependent manner [9,10] with different phenotypes depending on the developmental stage and duration of the exposure to ethanol [11,12]. Despite of that, the exact impact of these changes at the molecular and cellular levels is not fully understood. Furthermore, the genes of *C. elegans* and humans present 60–80% homology [13] and at least 80% homology of their proteome [14], and the resemblance between the nervous systems of the two species can clearly be highlighted. Dopaminergic as well as serotonergic neurons are present in their central nervous systems and play a key role in the reward system in the context of addiction and substance tolerance in humans and rodents, affecting it movement and behavior [15,16]. Dopaminergic [17–19] and serotonergic [20] neurotransmission have been involved in the control of responsiveness to ethanol and ethanol-induced behaviors. Nevertheless, the extent to which these mechanisms are conserved in worms is unclear [8,14,21].

In this study we aim to elucidate the mechanisms underlying the effects of extended 24-hour ethanol consumption in worms. To ascertain the impact of ethanol consumption on the worms’ motor capacity, we conducted a 3-step modified version of a kinetic chemotaxis assay (“diacetyl race”) [22] using worm populations at three different degrees of ethanol pre-exposure (no pre-exposure, medium, and high doses). By implementing this assay, we found distinct effects in wild-type, dopamine receptor mutant, and serotonin biosynthesis null mutant animals. Moreover, ethanol consumption changed the vesicle exocytosis of dopamine and serotonin from neurons and dysregulated the expression of a group of genes related to stress response. Furthermore, we demonstrate that the lifespans of wild-type, dopamine receptor mutant, and serotonin biosynthesis null mutant worms are all affected differently by long-term exposure to ethanol.

Finally, we offer a 3-step drug tolerance experimental model that is based on the established behavioral paradigm of the kinetic chemotaxis assay (“diacetyl race”) and can be used to test the levels of response to drugs other than ethanol in *C. elegans*. Tolerance assays have been proposed in previous studies for specific drugs and/or time-frames [23–25]. Nevertheless, here we aim to define a simple and reproducible tolerance assay that will pave the way for testing different compounds after different exposure times.

## Materials and methods

### Strains and transgenic lines

We followed the standard procedures for maintenance, genetic crosses, and other genetic manipulations of *C. elegans* [26]. In brief, all worm strains were cultivated on Nematode Growth Media (NGM) agar plates with *Escherichia coli* strain OP50 as the food source. The rearing temperature was maintained at 20°C to ensure optimal growth conditions unless otherwise specified. Throughout the rearing period, plates were routinely inspected for fungal contamination to ensure the exclusion of pathogen-exposed worms. The strains used in this study are listed in **Table 1** [27].

**Table 1:** Experimental models: *C. elegans.*

Strain	Genetic background	Source
N2: wild-type	Wild-type	*Caenorhabditis* Genetics Center (CGC)
BZ873: *dop-3 (ok295):* it lacks the D2-like receptor gene product (DOP-3).	This strain has been backcrossed with N2 (outcrossed).	*Caenorhabditis* Genetics Center (CGC): https://cgc.umn.edu/strain/BZ873
MT14984: *tph-1(n4622)*: it is defective for the gene encoding tryptophan hydroxylase, the rate-limiting enzyme in serotonin synthesis, leading to impaired serotonin biosynthesis.	This strain has been backcrossed with N2 (outcrossed).	*Caenorhabditis* Genetics Center (CGC): https://cgc.umn.edu/strain/MT14984
CL2166:dvIs19[pAF15(gst-4::GFP::NLS)]. (Oxidative stress-inducible GFP).	This strain has been backcrossed with N2 (outcrossed).	*Caenorhabditis* Genetics Center (CGC): https://cgc.umn.edu/strain/CL2166
P_tph-1_SNB: SEpHluorin (referred to as serotonin superecliptic strain #3001). Based on [27].	This strain has been generated by our group in N2 background.	Kindly provided by Eirini Lionaki and Persefoni Fragkiadaki
pro_asic_-1SNB: SEpHluorin (referred to as dopamine superecliptic strain #1785). Based on [27].	This strain has been generated by our group in N2 background.	Kindly provided by Dionysia Petratou

### Ethanol treatments

Animals were exposed to ethanol from the larval 4 (L4) stage through day 1 (D1) of adulthood. Age-matched worms (L4) were collected in suspension and distributed in low-dose ethanol-infused plates, high-dose ethanol-infused plates, and standard control plates. All plates contained 12 ml of NGM, with ethanol-infused plates prepared by adding 140 μl (low dose: 200 mM) or 280 μl (high dose: 400 mM, based on [22,28]) of pure ethanol into the NGM mixtures immediately before plate pouring, according to commonly used protocols for substance addition to NGM medium [29]. NGM plates were freshly prepared each time and covered with parafilm to minimize ethanol evaporation. Furthermore, all plates were sealed with parafilm during the 24-hour exposure period. All ethanol treatments were performed in the presence of UV-irradiated OP50 bacteria to reduce metabolization of ethanol by *E.coli*. Bacteria were exposed to UV irradiation for 30 minutes, in order to induce a viable but nonculturable state.

### Lifespan assays

Lifespan assays were conducted at 20°C unless otherwise specified. Synchronous animal populations were generated by hypochlorite treatment of gravid adults, yielding tightly synchronized embryos that were allowed to develop into adulthood under defined conditions. Once the worms reached the L4 stage, 20–25 individuals were transferred onto NGM plates seeded with UV-irradiated OP50 *E.coli* bacteria with/without ethanol, depending on the experimental group. After 24 hours of ethanol exposure, worms were washed for 1 hour with M9 and the experiment continued in NGM plates seeded with UV-irradiated OP50 *E.coli* bacteria without ethanol. A total of 150–200 animals were tested per condition in each experiment. Worms were transferred to fresh plates every 2 days and monitored daily for touch‐provoked movement and pharyngeal pumping until death. Worms that died due to internally hatched eggs, extruded gonads, or desiccation from crawling off the edge of the plates were censored and recorded as such in the dataset. Each survival assay was repeated three times, and the figures represent typical assays. Survival curves were generated using the Kaplan-Meier product‐limit method, and the log‐rank (Mantel–Cox) test was used to assess differences between survival curves and determine *p*‐values. Statistical analysis and lifespan calculations were performed using the Prism software package (GraphPad Prism 8). The median survival time is defined as the time point at which the probability of survival equals 50%, meaning that the survival curve crosses 50% survival (**S1 Table**).

### Motility Assay: Thrashing

The thrashing, or swimming assay, is a sensitive method for detecting locomotion defects and assessing the overall health status. In this assay, worms are treated with/without ethanol for 24 hours, washed with M9 for 1 hour and then placed in a small NGM Petri dish overlaid with a physiological buffer (M9), and their average swimming speed is measured. The thrashing analysis was conducted by videotaping the worms in the thrashing buffer for 2 minutes using a Zeiss Lumar V12 SteREO microscope at 20x magnification after treatment with the corresponding dose of ethanol (0 mM, 200 mM, 400 mM or 600 mM) and subsequent 1-hour post-exposure withdrawal period (M9 buffer). The video files were then analyzed using Wormlab software (version 2022.1.1). The software semi-automatically detects the worms within the field of view and calculates various statistics based on the movement patterns of the worms. According to the Wormlab software manual, swimming metrics are defined as follows: “wave initiation rate” refers to the number of body waves initiated from either the head or tail per minute, “brush stroke” refers to the area traced by the worm’s body during a single complete stroke, and “activity” represents the brush stroke normalized by the time taken to perform two strokes.

### Kinetic chemotaxis assay (“diacetyl race”)

Exposure was carried out as described previously (**Ethanol Treatments** section). Following the initial exposure period (24 hours), worms were washed off the corresponding plates [22], repeatedly rinsed, and suspended in M9 buffer in Eppendorf tubes for the so called 1-hour post-exposure withdrawal period. The setup and execution of the kinetic chemotaxis assay were adapted from [5]. Standard chemotaxis assay plates were prepared in 10 cm diameter Petri dishes with 15 ml chemotaxis agar medium (a modified NGM medium without peptone), while re-exposure chemotaxis assay plates were infused with a low dose of ethanol (60 mM), a concentration where motility and feeding behaviors are minimally affected, in order to minimize confounding factors and based on previous studies [30]. All chemotaxis assay plates were freshly prepared within 24 hours of a kinetic chemotaxis assay to ensure consistent and precise ethanol concentrations. The bottoms of the plates were marked at the two opposite poles, indicating a starting field and a goal area behind a finish line, with each field covering approximately 5 cm^2^ of the plate. Immediately before the assay, a 10 μl mixture of attractant (1:1000 diacetyl, chemically known as 2,3-butanedione) and paralytic (100 mM sodium azide) was applied to the goal. The suspended worms were allowed to naturally settle at the bottom of the Eppendorf tubes, after which most of the buffer solution was drained to facilitate the collection of a concentrated drop of worms via pipette. Drops containing 25–60 worms were pipetted onto the starting fields of chemotaxis assay plates. To remove excess fluid around the worms, gentle manual absorption with single-layered tissue paper was applied where necessary. Three independent kinetic chemotaxis assays were conducted. The total number of worms and the number of worms that reached the goal were counted every 10 minutes over the period of 60 minutes.

### Fluorescent microscopy

Fluorescence imaging of worms was conducted by mounting and paralyzing the nematodes with a 20 μl drop of 20 mM levamisole on standard microscope slides. All animals were age-synchronized (treatment at L4 and imaging at D1) to ensure uniform developmental stages, and photobleaching was minimized by single-exposure imaging per animal (5–10 worms/experimental condition were imaged in each session). Fluorescence imaging was conducted at room temperature using a Zeiss LSM 900 confocal microscope with an Airyscan 2 module. We used a 40x/1.2 NA Multi-immersion objective (LD LCI Plan-Apochromat) and set the pinhole to 1 Airy unit. Samples were excited with a 488nm laser, while acquisition parameters—including laser power, gain, and 2048 × 2048 resolution—were kept constant across all groups. Post-acquisition processing was performed in Zen Blue, and data analysis was carried out in ImageJ/Fiji. All analyses were conducted by an investigator who was blinded to group assignment during image evaluation.

### Image analysis

To accurately represent the biological signal and account for the technical detector offset of the Airyscan 2 system (baseline of approximately 10,000 units), we performed background subtraction. The mean background intensity was calculated from non-fluorescent regions and subtracted from all raw measurements (Mean_adjusted_ = Mean_raw_ - Background). All image quantifications were performed using ImageJ/Fiji.

### mRNA quantification

Total RNA was extracted using TRIzol reagent, following the manufacturer’s protocol. RNA from 150 worms per condition was collected after pre-exposure (24 hours) with the corresponding concentration of ethanol and 1-hour post-exposure withdrawal period. RNA concentration was measured using a NanoDrop Microvolume Spectrophotometer before performing cDNA. For cDNA synthesis, 1 μg mRNA was reverse transcribed using the iScriptTM cDNA Synthesis Kit (BioRad). Quantitative polymerase chain reaction (qPCR) was performed with the Eva Green qPCR Kit (Biotium) according to the manufacturer’s instructions. The qPCR reactions were run on a Bio‐Rad CFX96 Real‐time PCR system. All the primers used are listed in **Table 2**. *act-1* served as the housekeeping gene. mRNA expression across samples was normalized using the ΔΔCt (Delta-Delta Ct) method [31]. First, data were normalized to the *act-1* mRNA levels of each condition to obtain ΔCt values, and then mRNA levels were normalized to the appropriate control reference conditions.

**Table 2:** Oligonucleotides.

Gene target	Forward primer (5’— 3’)	Reverse primer (5’— 3’)
*act-1*	AGGCCCAATCCAAGAGAGGTATC	TGGCTGGGGTGTTGAAGGTC
*skn-1*	TCCACCAGGATCTCCATTCG	CTCCATAGCACATCAATCAAGTCG
*sod-3*	ATTGCTCTCCAACCAGCGC	GGAACCGAAGTCGCGCTTAA
*daf-2*	AGCTCTCGGAACAACCACTG	TGACAAGTCGAAGCCGTCTC
*daf-16*	GCGAATCGGTTCCAGCAATTCCAA	ATCCACGGACACTGTTCAACTCGT
*adh-1*	GGAAAGAATGTTACTGGATGGCA	ATTCGCAGTTGAGGCAGTTG
*gst-4*	ATGGTCAAAGCTGAAGCCAACG	CTGCAGTTTTTCCAGCGAGTCC

### Quantification and statistical analysis

Quantification was performed using ImageJ software (NIH, http://imagej.nih.gov/ij/) and Volocity 6.3 software. Statistical analyses were conducted using the Prism software package (GraphPad Software Inc., San Diego, USA) and Microsoft Office 2010 Excel (Microsoft Corporation, Redmond, WA, USA). The specific statistical tests applied for each experiment are described in Figure legends. Results for continuous variables are expressed as mean and standard deviation in all plots. For all experiments, at least three independent biological replicates were performed unless specified otherwise. For the kinetic chemotaxis assay (“diacetyl race”), each experiment was conducted in tandem sets of two kinetic chemotaxis assays, and the average percentage of worms reaching the goal was calculated for each time point. The Shapiro–Wilk normality test was used to assess normality. Statistical significance between two groups was determined using Student’s t test (p < 0.05 unless otherwise specified). Statistical significance for more than two groups was assessed by 1-way ANOVA (p < 0.05 unless otherwise specified). 2-way ANOVA was used to test the interaction of two factors (**S2 Table**). In all cases, the statistical test that has been applied is specified in the corresponding Figure legend. Multiple comparisons were corrected using the Benjamini–Hochberg false discovery rate (FDR) post hoc test to control for type I error.

## Results

### A 24-hour exposure to ethanol reduces lifespan of wild-type worms but not of dopamine receptor and serotonin synthesis mutants

Firstly, to assess any long-term consequences of the treatment, we conducted survival assays following 24 hours of treatment with low (200 mM) and high (400 mM) ethanol concentrations, starting from the L4 stage to day 1 (D1) of adulthood. Previous studies have shown that exposure of worms to 500 mM ethanol resulted in an internal ethanol (EtOH) concentration relevant to human consumption and disinhibition behaviour in rodents [19]. Nevertheless, this concentration extremely inhibits crawling [32] and we thereby decided to perform our experiments at lower doses. We selected 400 mM ethanol, which has been previously tested by others [18,22], and included a second condition where we observed mild effects in locomotion (200 mM).

**Fig 1:**
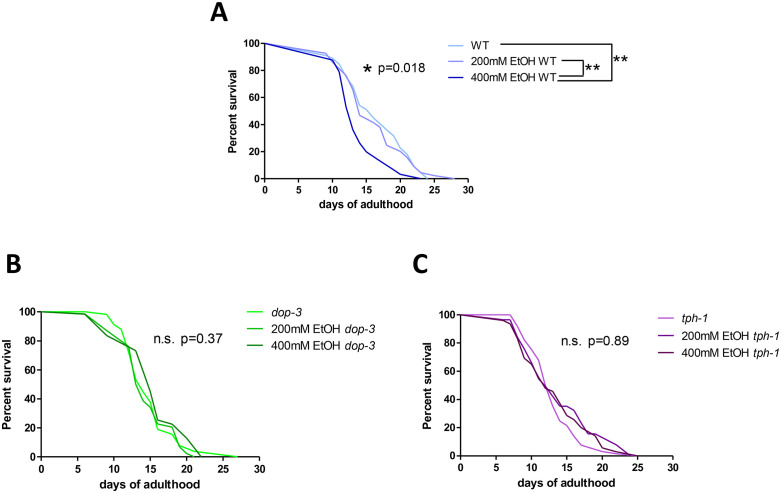
Exposure to high doses of ethanol affects the lifespan of wild-type worms but not *dop-3* and *tph-1* mutants. Survival curves of **(A)** wild-type (N2 strain) animals, and **(B)** dopamine receptor (*dop-3(ok295)*) and **(C)** serotonin synthesis (*tph-1(n4622)*) mutant strains from day 1 (D1) of adulthood, immediately after 24-hour treatment with 200 mM ethanol, 400 mM ethanol or no treatment. P-values were calculated by Log-rank/Mantel–Cox for each graph and indicated next to the curve: *p < 0.05, n.s.; non-significant. Log-rank/Mantel–Cox test was also performed for the comparison of each pair of conditions and only statistically significant comparisons are shown: **p < 0.01.

The lifespan of wild-type N2 worms did not change in response to 200mM ethanol, but significantly decreased after 24 hours of treatment with the higher dose (400mM) of ethanol (**Fig 1**, **S1 Table**). As a proof-of-concept approach we decided to test two strains with different, complete and partial deficits in serotonergic and dopaminergic neurotransmission; a dopamine receptor null mutant *dop-3 (ok295)* [33] and the serotonin biosynthesis null mutant *tph-1(n4622)* [34] strains. In our experiments, the basal lifespans of both the *dop-3* and *tph-1* mutant strains were reduced compared to wild-type (**S1 Fig**). However, their lifespan was not further decreased by 200- or 400-mM ethanol. Thus, a lifespan-shortening effect of ethanol exposure was observed in wild-type animals but was not detected in *dop-3* or *tph-1* mutants under the conditions tested.

### A 24-hour ethanol exposure does not affect worm motor activity

**Fig 2:**
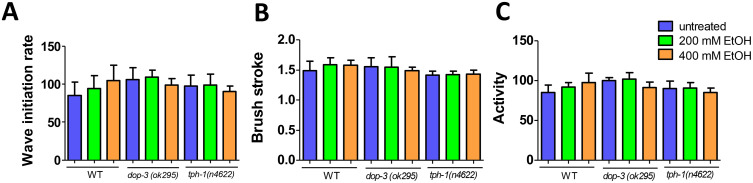
A 24-hour ethanol exposure does not affect the swimming behavior of adult worms. Panels plot worm locomotion data of **(A)** wild-type (N2 strain) animals, and **(B)** dopamine (*dop-3(ok295)*) and **(C)** serotonin (*tph-1(n4622)*) mutant strains from day 1 (D1) of adulthood, after 24 hours-treatment with 200 mM ethanol, 400 mM ethanol or no treatment, and 1 hour of post-exposure withdrawal period. Mean and standard deviation are depicted for each group. P-values were calculated by T-test corrected using the Benjamini–Hochberg false discovery rate (FDR) post hoc test (N = 3 independent experiments).

After assessing the long-term effects of ethanol on the lifespan of the nematodes, the short-term impact of exposure was evaluated. For this purpose, we analyzed the motor behavior of our three strains (wild-type, dopamine mutants, and serotonin mutants) under three experimental conditions: no treatment, 24-hour incubation with 200 mM, and 24-hour incubation with 400 mM ethanol, followed by a 1-hour recovery period. We did not observe any significant differences in the parameters analyzed (**Fig 2**), indicating that the worms maintain movement after exposure.

However, as expected [10], higher doses of ethanol (600 mM), even after short exposure periods (20 minutes) reduced the movement of the nematodes (**S2 Fig**), although movement was not completely impaired. Specifically, the motor activity of wild-type worms and *dop-3* mutants was more affected than that of the *tph-1* mutants following acute ethanol exposure.

Therefore, since worms preserve their locomotion after an extended exposure to 200 and 400 mM ethanol, we employed these concentrations in further studies.

### Intact dopamine and serotonin neuronal circuits are required for tolerance to ethanol

**Fig 3:**
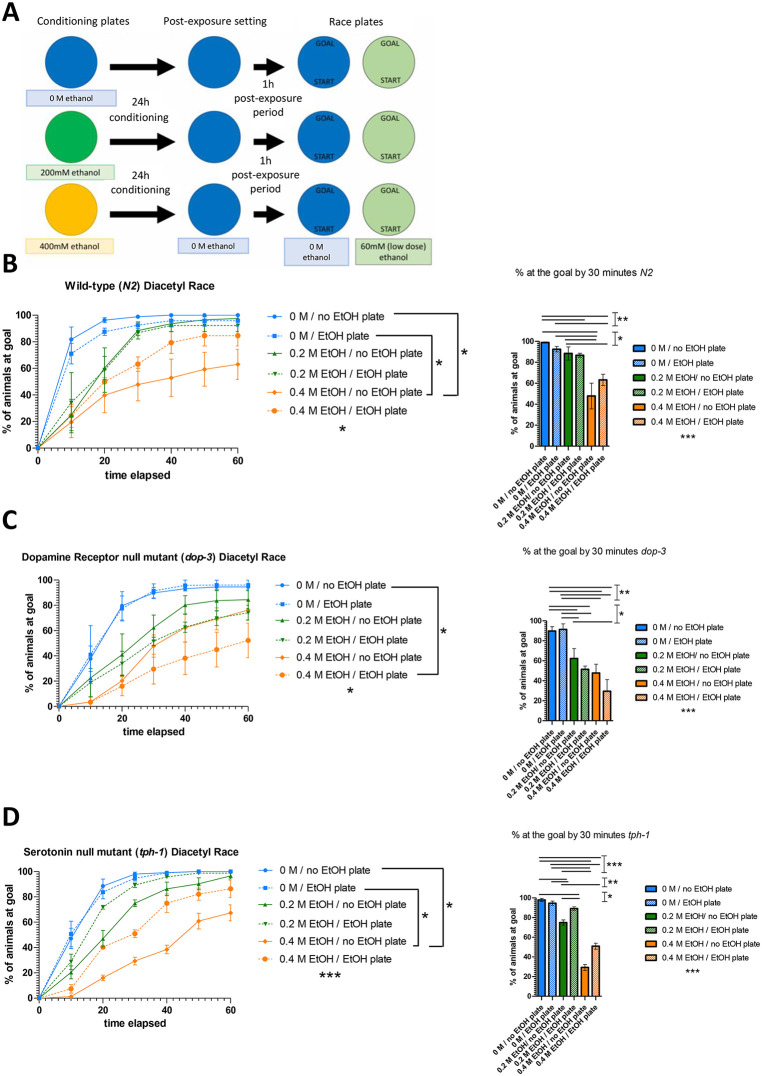
Intact dopamine and serotonin neuronal circuits are required for tolerance to ethanol (A) Schematic diagram of the kinetic chemotaxis assay (“diacetyl race” paradigm). For the “pre-exposure” period, larval 4 (L4) stage worms are subjected to no treatment or treatment with 200 mM of ethanol, or 400mM of ethanol for 24 hours (until day 1 of adulthood, D1). After the “pre-exposure” period, all the worms were removed from the ethanol infused environment to simulate a post-exposure withdrawal setting for 1 hour. After withdrawal, worms of each pre-exposure group were divided into standard “no EtOH” kinetic chemotaxis assay plates or 60 mM ethanol infused “EtOH” kinetic chemotaxis assay plates, all of them marked with a start and finish line. Performance of **(A)** wild-type (wild-type, N2 strain) animals, **(B)** dopamine (*dop-3(ok295)*) and **(C)** serotonin (*tph-1(n4622)*) mutant strains are depicted. P-values were calculated by 1-way ANOVA for all conditions (indicated below the legend) and by T-test for pairwise comparisons corrected using the Benjamini–Hochberg false discovery rate (FDR) post hoc test (at least N = 3 independent experiments): *p < 0.05, **p < 0.01, ***p < 0.001, ****p < 0.0001. The results of 2-way ANOVA are shown in S2 Table.

Next, we investigated the tolerance of worms to ethanol and the subsequent effects on sensorimotor performance using a 3-step modified version of a kinetic chemotaxis assay (commonly known as “diacetyl race”) (**Fig 3A**, see **Methods**). Briefly, the assay consists of 1) 24-hour exposure to 0 mM, 200 mM or 400 mM ethanol, 2) 1-hour post-exposure period (cessation of alcohol intake), and 3) evaluation of the worms’ capacity to reach a goal with or without re-exposure to ethanol (60 mM) during the chemotaxis assay. Importantly, the 1-hour post-exposure period has been defined as withdrawal since internal ethanol concentration drop have previously demonstrated in the same experimental setup [5]. Based on previous studies showing that motility and feeding behaviors are minimally affected at 60 mM ethanol, this concentration was selected for the kinetic chemotaxis assay [5,30]. Nevertheless, the output of the chemotaxis assay is defined by multiple parameters that are affected by ethanol, such as sensory perception, neural integration or decision making, reward and motor coordination. We found that the performance of wild-type animals was not impaired following primary exposure to 60 mM ethanol (**Fig 3B**, blue). Notably, the performance of these wild-type *C. elegans* exposed for 24 hours to a high dose of ethanol (400 mM), and which then underwent withdrawal, was recovered upon re-exposure to ethanol (**Fig 3B**, orange, condition “0.4 M EtOH/EtOH plate” does not differ from the “0 M” conditions, whereas “”0.4 M EtOH/no EtOH plate” does), suggesting a mechanism not related to ethanol toxicity, but a state- and experience-dependent change in behavior. In contrast to the wild-type nematodes, dopamine and serotonin mutant strains exhibited reduced basal chemotaxis speed, which did not change in response to 60 mM ethanol (Fig 3B-3D, blue, and **S3A Fig**). Interestingly, re-exposure to ethanol exacerbated the 1-hour post-exposure period withdrawal effects in *dop-3* worms pre-conditioned with both 200 mM and 400 mM ethanol (Fig 3C, green and blue) while ameliorating the impaired chemotaxis of *tph-1* worms (Fig 3D, green and blue). Analysis of each treatment condition separately (**S3 Fig**) revealed genotype-specific effects on performance in the kinetic chemotaxis assay (“diacetyl race”) in most cases. Notably, differences were observed in groups pre-conditioned with 200 mM or 400 mM ethanol followed by re-exposure. Interestingly, re-exposure to 60 mM promoted a decline in the percentage of *dop-3* worms reaching the goal at 30 minutes, which did not occur in wild-type and *tph-1* worms (**S3B Fig**), suggesting the possibility that signalling through the dopamine D2-like receptor is required for the maintenance of chemotaxis to diacetyl both after recovery and re-exposure. Taken together, these findings suggest that dopaminergic and serotonergic neurons are involved in the mechanisms underlying ethanol tolerance.

### Ethanol consumption increases neuronal dopamine and serotonin vesicle exocytosis

**Fig 4:**
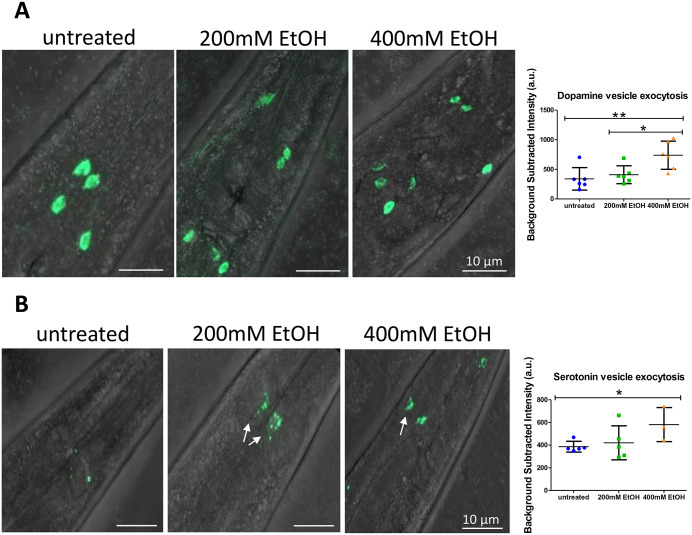
Ethanol consumption increases dopamine and serotonin vesicle exocytosis in worm neurons. Representative images of the proximal worm body, using day 1 (D1) adult animals of the **(A)** 1785[proasic-1SNB::SEpHluorin] and the **(B)** 3001[Ptph-1SNB::SEpHluorin] strains, treated with 200 mM ethanol, 400 mM ethanol or no treatment for 24 hours prior to imaging (40x objective lens). Mean fluorescence (a.u.; arbitrary units) and standard deviation are depicted for each group. P-values were calculated by 1-way ANOVA for all conditions (**p < 0.01 for graph [A] and non-significant for graph **[B]**) and by T-test for pairwise comparisons corrected using the Benjamini–Hochberg false discovery rate (FDR) post hoc test (at least N = 3 independent experiments): *p < 0.05, **p < 0.01.

To investigate the cellular mechanisms underlying the differential performance of each strain in the chemotaxis assay, we employed two *C. elegans* strains in which dopamine and serotonin vesicle fusion can be quantified via pH-dependent green fluorescent protein (GFP) fluorescence dequenching [35]. These strains express a variant of the Rosella biosensor, consisting of a tandem pH-sensitive GFP (pHluorin/pHn) under the control of specific dopamine or serotonin promoters. The promoter of *asic-1* and the *tph-1* promoter drive expression of GFP upon vesicle secretion in the dopaminergic neurons (CEPD, CEPV, ADE, and PDE) and the serotonergic neurons (NSM and ADF), respectively (see **Methods**) [27,36]. Therefore, these strains report changes in vesicle exocytosis rather than neurotransmitter concentration or release, since vesicles with different transmitter contents would produce identical pHluorin signals. Our results demonstrate that consumption of high doses of ethanol (400 mM) leads to increased dopamine and serotonin vesicle exocytosis in nematode neurons (**Fig 4**). Specifically, serotonergic NSM neurons seem to be the primary neurons increasing vesicle exocytosis upon treatment with 200 mM ethanol, whereas ADF neurons may display enhanced vesicle exocytosis at the concentration of 400 mM. Notably, dopamine vesicle exocytosis was elevated upon treatment with both doses of ethanol, whereas serotonin secretion was significantly increased only following treatment with the higher ethanol dose (400 mM). These findings may suggest that the activation of dopaminergic and serotonergic neuronal circuits may contribute to the effects of ethanol observed in the chemotaxis assay. Nevertheless, due to the limitation of the reporter strains not quantifying dopamine and serotonin release but vesicle exocytosis, these findings should be interpreted cautiously, as identical pHluorin signals can arise from vesicles with different transmitter contents.

### Dopaminergic and serotonergic circuits modulate ethanol-induced changes in stress gene transcription

To further elucidate the molecular mechanisms underlying the results obtained in our chemotaxis and lifespan assays, we assessed the expression levels of genes involved in stress responses, particularly oxidative stress and ethanol metabolism. We first examined the role of the master regulator of xenobiotic, oxidative and pathogen stress responses, SKN-1 (a transcription factor orthologue to mammalian Nrf2 that is capable of activating detoxification responses), by utilizing a *gst-4* transcriptional reporter strain, as *gst-4* (encoding glutathione S-transferase, involved in oxidative stress responses) is a key target gene of SKN-1 that mediates antioxidant/detoxification responses (**S4 Fig**). Following 24-hour treatment with 200 mM ethanol, no statistically significant changes in *gst-4:GFP* expression were observed. Unfortunately, the general health status of the GST-4::GFP worms was widely affected at 400 mM EtOH dose, precluding any analysis.

**Fig 5:**
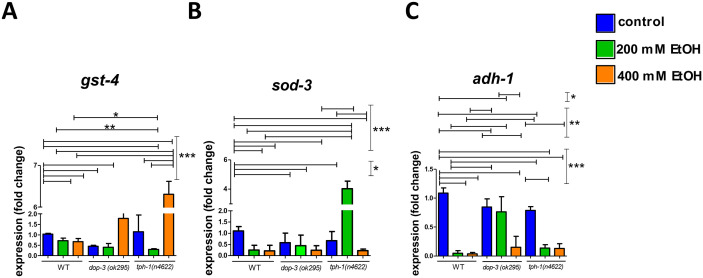
Dopaminergic and serotonergic circuits modulate ethanol-induced changes in stress gene transcription. Gene expression of **(A)** *gst-4*, **(B)** sod-3 and **(C)** *adh-1* in wild-type worms, and dopamine (*dop-3(ok295)*) and serotonin (*tph-1(n4622)*) mutant strains treated with 200 mM ethanol, 400 mM ethanol or no treatment for 24 hours. Standard deviation is depicted for each group. For qPCR, p-values were calculated by 1-way ANOVA for all conditions: p < 0.001 was obtained for *gst-4*, *sod-3* and *adh-1*. P-values were calculated by T-test for pairwise comparisons corrected using the Benjamini–Hochberg false discovery rate (FDR) post hoc test (at least N = 6 independent experiments): *p < 0.05, **p < 0.01, ***p < 0.001.

To gain a more comprehensive understanding of the genes implicated in the effects of ethanol, and the differential performance between wild-type and dopamine- and serotonin-deficient mutants in the chemotaxis assay, we quantified the expression of *gst-4* (**Fig 5A**), *sod-3* (a gene encoding superoxide dismutase, also involved in oxidative stress responses and a target of DAF-16, a master regulator of longevity and metabolic, oxidative, pathogen stress responses) (**Fig 5B**), and *adh-1* (encoding the enzyme alcohol dehydrogenase 1, involved in ethanol metabolism) (**Fig 5C**) using qPCR. Notably, both DAF-16 [37] and ADH-1 [38] were found to play a role in lifespan regulation. Reducing *daf-2* activity allows *daf-16* to promote longevity, whereas ADH-1 exerts its effect on lifespan at least partially via metabolizing glycerol. All three genes were downregulated in wild-type nematodes following ethanol exposure, but their expression patterns were altered in *dop-3 (ok295*) and *tph-1 (n4622*) mutants. The expression of *gst-4* was upregulated upon 400 mM treatment in *dop-3 (ok295*) and *tph-1 (n4622*) mutants, in contrast to other 400mM-treated wild-type animals and to other treatments (**Fig 5A**). A remarkable upregulation of *sod-3* expression was observed in 200mM-treated *tph-1 (n4622*) worms, but not in 400mM-treated *tph-1 (n4622*) worms (**Fig 5B**). Possible explanations for this surprising phenomenon are tightly-regulated expression of *sod-3* expression at low ethanol concentrations, whereas higher ethanol concentrations activate other yet unknown mechanisms. In contrast, *adh-1* mRNA is downregulated upon ethanol exposure (**Fig 5C**). All together, these results suggest that these genes are affected by ethanol consumption.

## Discussion

**Fig 6:**
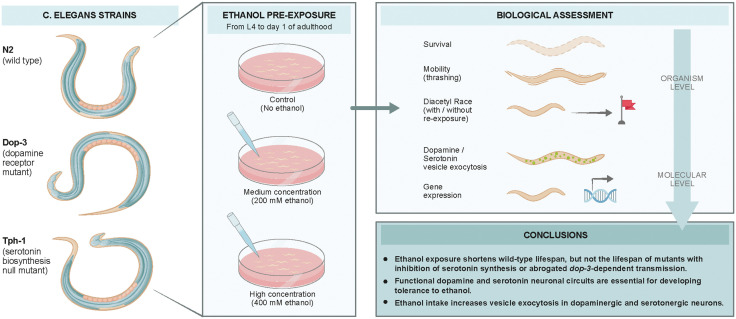
Summary of this study.

In the present study, we aimed to implement a multifaceted experimental approach to determine the effects of ethanol on *C. elegans* (**Fig 6**). According to our data, ethanol appears to act through dopamine- and serotonin-dependent neuromodulatory pathways and these pathways shape ethanol-related behaviour, lifespan and stress gene expression. The behavioural effects are dependent on exposure to ethanol (prior and present) and genotype.

Ethanol exposure at the L4 stage had a negative impact on the lifespan of wild-type worms. Furthermore, exposure to 400 mM ethanol impairs lifespan and the *tph-1* and *dop-3* genes contribute to the ethanol-induced shortening of lifespan (**Fig 1**, **S1 Fig**). A possible interpretation for this phenomenon is that ethanol may reduce lifespan through dopaminergic and serotonergic pathways, as dopamine and serotonin mutant strains seem to be resistant to the detrimental effects of ethanol observed in wild-type worms (**Fig 1**). Alternatively, the shorter lifespan of these mutants might prevent the appearance of detrimental late-life effects of ethanol. However, ethanol significantly impaired median lifespan in wild-type animals, indicating increased mortality already in early and midlife in the wild-type, which is entirely absent in the mutants (**Fig 1**, **S1 Table**). Moreover, lifespans of *dop-3* and *tph-1* mutants are comparable to those of ethanol-treated wild-type worms, showing that mutant strains live sufficiently long to exhibit ethanol-induced effects. While our data do not formally exclude the possibility that genotype-specific mortality masks ethanol effects in these mutants, the complete absence of ethanol-induced lifespan shortening in both strains is more consistent with a requirement for intact dopamine and serotonin signaling in mediating ethanol’s detrimental effects on longevity.

Importantly, according to the literature, the effects of ethanol are both time- and dose-dependent, with primarily beneficial effects observed at low doses during early developmental stages [12,39,40]. Indeed, the dual role of ethanol, with beneficial and harmful effects for a plethora of biological processes and depending on the dose and duration of the treatment, poses a challenge for the comparison and extrapolation of results obtained in different experimental set ups [41–43].

Remarkably, other *tph-1* loss-of-function mutations have been previously linked to increased (in the presence of FUDR, which we do not use in the present study) [44,45], unchanged [44,46] or decreased lifespan [47], in comparison to wild-type. Nevertheless, these results were found in slightly different experimental setups and *tph-1* lifespan is sensitive to availability of food and FUDR treatment. In any case, the lifespan reduction in *tph-1* mutants [47] is in accordance with our data (**Fig 1**, **S1 Fig**), and we can speculate that *tph-1*- related lifespan reduction precludes a further detrimental effect of alcohol treatment. Moreover, longevity of *dop-3* mutants has been assessed in other studies, either showing non-significant changes compared to wild-type [48], or results that cannot be extrapolated and compared to our findings due to the lack of appropriate wild-type controls [49–51].

We also found that acute ethanol exposure affected the sensorimotor performance of *C. elegans* during the chemotaxis assay, as evidenced by differences in behaviour between worms on post-exposure withdrawal plates and those on ethanol plates (**Fig 3**, **S3 Fig** and **S2 Table**). Notably, re-exposure to ethanol exacerbated the withdrawal effect in *dop-3* mutants in a manner similar to the wild-type nematodes, but with more pronounced impairment. In contrast, re-exposure improved the impaired locomotion performance in *tph-1* mutants, indicating that serotonin plays a crucial role in mediating the effects of ethanol. In our study, worms retained movement after ethanol treatment, and basal movement seems to be comparable across strains (as shown by values for the untreated worms in **Fig 2** and **S2 Fig**). Nevertheless, their behavior during the chemotaxis assay was characterized by uncoordinated and “untargeted” movement, suggesting a disruption in motor coordination, orientation or chemoattraction towards the goal. Notably, ethanol exposure has been shown to alter multiple aspects of nervous system function and whole-body physiology in animal models, including worms [5]. Importantly, outcomes in the kinetic chemotaxis assay (“diacetyl race”) are shaped by parameters such as neural integration, decision making, reward, locomotion and orientation, which should be considered when interpreting the results [52,53].

Many studies have shown that ethanol affects a wide range of behaviors of worms and that this regulation is, at least partly, mediated by different components of the serotonergic and dopaminergic pathways [17–20]. While some behaviors are inhibited (for example headbend frequency), others are disinhibited (such as touch- and light-response, crawling speed, and a behaviour called “foraging” that is associated with feeding) in wild-type animals upon ethanol consumption and specific factors involved in dopamine synthesis and D1-like dopamine signaling (such as *dop-1* and *dop-4*) are required for these behaviors [19]. Despite significant differences in the experimental setup and the worm strains used, our findings are in line with this study, since in our chemotaxis assay we observed disruption of *dop-3* behavior after the 60 mM re-exposure, suggesting the involvement of the D2-like receptor in loss of motivation. We also found different gene expression patterns of SKN-1 and DAF-16 targets, as well as *adh-1*, although further studies are needed to disentangle the role of dopaminergic and serotonergic signalling in the regulation of the metabolic network of alcohol detoxification.

A central aspect of our study is the 3-step experimental design (24 hour-drug [i.e., ethanol] exposure, 1-hour post-exposure withdrawal, chemotaxis assay) (**Fig 3**), a behavioral assay developed to assess the effects of previous exposure to a specific drug (i.e., ethanol, in our study) in the navigational efficiency of *C. elegans* in a controlled setting. Our 3-step tolerance experimental set-up can potentially be used to evaluate both the immediate and long-term effects of ethanol exposure, including post-exposure withdrawal responses and potential recovery of navigational abilities upon re-exposure. Of note, tolerance assays specifically tailored to the unique physiological traits of nematodes have been previously proposed for specific drugs and/or time frames [23–25]. Nonetheless, our 3-step experimental design is particularly valuable because it simulates behavioural paradigms commonly observed in higher vertebrates and it is versatile, i.e., it can be used for testing tolerance to a plethora of drugs at different time points.

We employed different methods to assess ethanol-induced effects on *C. elegans*, including molecular changes and behavioral outcomes. *C. elegans* is as an exemplary model for studying tolerance-related behavior due to its simplicity, ease of genetic manipulation, and the extensive knowledge of its nervous system. Although further research is needed, this study demonstrates the strengths of *C. elegans* to explore the complex interplay between ethanol exposure and tolerance-related pathways, contributing valuable insights to the broader field of alcohol research and paving the way for new intervention strategies.

## Conclusions

Three worm strains (wild-type, a dopamine D2-like receptor mutant and a serotonin biosynthesis null mutant) exhibit different responses to ethanol in various assays. Indeed, ethanol consumption seems to alter neuronal vesicle exocytosis of dopamine and serotonin, as well as the expression of a panel of genes associated with stress responses. Additionally, the lifespan of wild-type and mutant worms is affected differently by extended (24-hour) ethanol exposure. Importantly, our study takes a proof-of-concept approach, focusing on comparing two mutant strains with wild-type worms, rather than conducting a comprehensive mutation screen. Future studies exploring additional perturbations of the dopamine and serotonin pathways will be important to further validate and expand these findings. Our findings offer new insights into alcohol consumption, with a focus on the roles of dopaminergic and serotonergic neurotransmission. Importantly, our kinetic chemotaxis assay (“diacetyl race”) model has potential for investigating the effects of other addictive substances beyond alcohol.

### Practical applications

Our findings on how ethanol impacts lifespan, movement patterns, and cellular and genetic structures, pave the way for future studies shading light on mechanisms underlying ethanol-induced neuroplasticity. Therefore, here we enhance our understanding of the biological foundations of ethanol consumption, which may ultimately lead to potential therapeutic targets for the treatment of alcohol use disorders in humans.

## Supporting information

S1 Fig Lifespan of *dop-3* and *tph1* mutants is reduced in basal conditions compared to wild-type animals and exposure to ethanol differentially affects the lifespan of each strain. Survival curves of wild-type (N2 strain) worms and dopamine (*dop-3(ok295)*) and serotonin (*tph-1(n4622)*) mutant strains from day 1 (D1) of adulthood, in **(A)** basal conditions (no treatment), immediately after 24 hours-treatment with **(B)** 200 mM ethanol or **(C)** 400 mM ethanol. P-values were calculated by Log-rank/Mantel–Cox for each graph and indicated next to the curve: *p < 0.05, ***p < 0.001, n.s.; non-significant. Log-rank/Mantel–Cox test was also performed for the comparison of each pair of conditions and only statistically significant comparisons are shown: **p < 0.05, **p < 0.01, ***p < 0.001.
(PPTX)

S2 Fig Short-term treatment with high doses of ethanol alters the swimming behavior of adult worms. Panels plot worm locomotion data of wild-type (N2 strain) animals, and dopamine (*dop-3(ok295)*) and serotonin (*tph-1(n4622)*) mutant strains from day 1 (D1) of adulthood, after 20 minute-treatment with 600 mM ethanol or no treatment, and 1 hour of post-exposure withdrawal period. Mean and standard deviation are depicted for each group. P-values were calculated by T-test corrected using the Benjamini–Hochberg false discovery rate (FDR) post hoc test (N = 5 independent experiments) *p < 0.05, **p < 0.01, ***p < 0.001.
(PPTX)

S3 Fig The performance on the kinetic chemotaxis assay (“diacetyl race”) is affected by the genotype of the worms. Performance of wild-type (wild-type, N2 strain) animals, dopamine (*dop-3(ok295)*) and serotonin (*tph-1(n4622)*) mutant strains are depicted for each experimental condition **(A)** during the whole kinetic chemotaxis assay (“diacetyl race”) and **(B)** at 30 minutes. P-values were calculated by 1-way ANOVA for all conditions (indicated next to the curve/ bar graph) and by T-test for pairwise comparisons corrected using the Benjamini–Hochberg false discovery rate (FDR) post hoc test (at least N = 3 independent experiments): *p < 0.05. The results of 2-way ANOVA are shown in S2 Table.
(PPTX)

S4 Fig Representative images and quantification of mean fluorescence (a.u.; arbitrary units) of the *gst-4* transcriptional reporter strain day 1 (D1) adult animals treated with 200 mM ethanol or no treatment for 24 hours prior to imaging (40x objective lens). No statistically significant differences were detected using a T-test corrected using the Benjamini–Hochberg false discovery rate (FDR) post hoc test.
(PPTX)

S1 Table Data from the independent lifespan experiments. (PPTX)

S2 Table Results of 2-way ANOVA analysis for Fig 3 and S3 Fig. (DOCX)

S1 File Raw data. (XLSX)
