# Supplementary figures and images for: Specific genes of the dopaminergic (dop-3) and serotonergic (tph-1) pathways contribute to the effects of ethanol consumption in Caenorhabditis elegans
Source: PLoS One. 2026 Mar 23;21(3):e0344966. doi: 10.1371/journal.pone.0344966 (PMC13008063; doi:10.1371/journal.pone.0344966)

## Slide 1
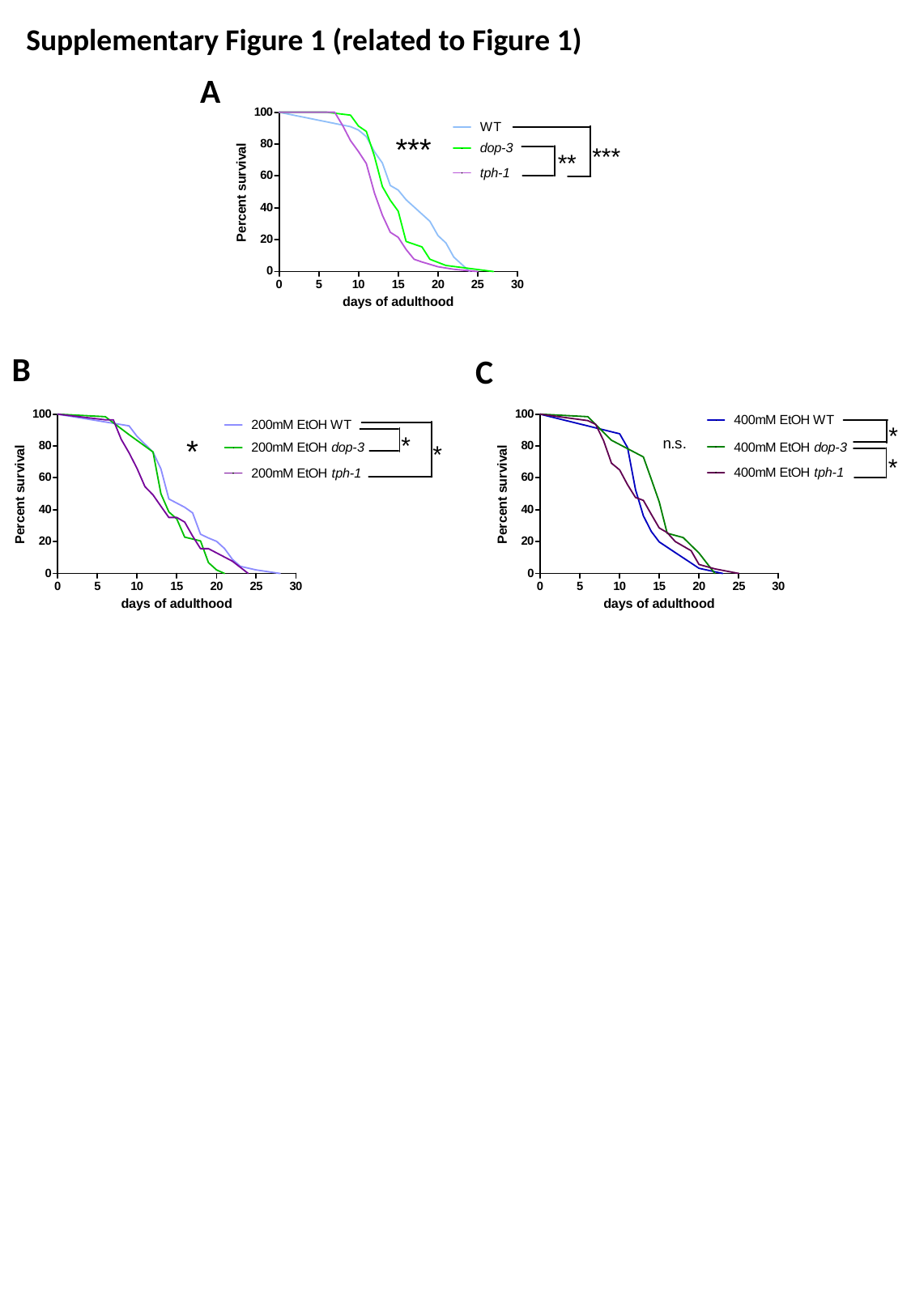

Supplementary Figure 1 (related to Figure 1)
A
B
C

Supplement: S1 Fig — Survival curves of wild-type (N2 strain) worms and dopamine (dop-3(ok295)) and serotonin (tph-1(n4622)) mutant strains from day 1 (D1) of adulthood, in (A) basal conditions (no treatment), immediately after 24 hours-treatment with (B) 200 mM ethanol or (C) 400 mM ethanol. P-values were calculated by Log-rank/Mantel–Cox for each graph and indicated next to the curve: *p < 0.05, ***p < 0.001, n.s.; non-significant. Log-rank/Mantel–Cox test was also performed for the comparison of each pair of conditions and only statistically significant comparisons are shown: **p < 0.05, **p < 0.01, ***p < 0.001. (PPTX) [file pone.0344966.s001.pptx]

## Slide 1
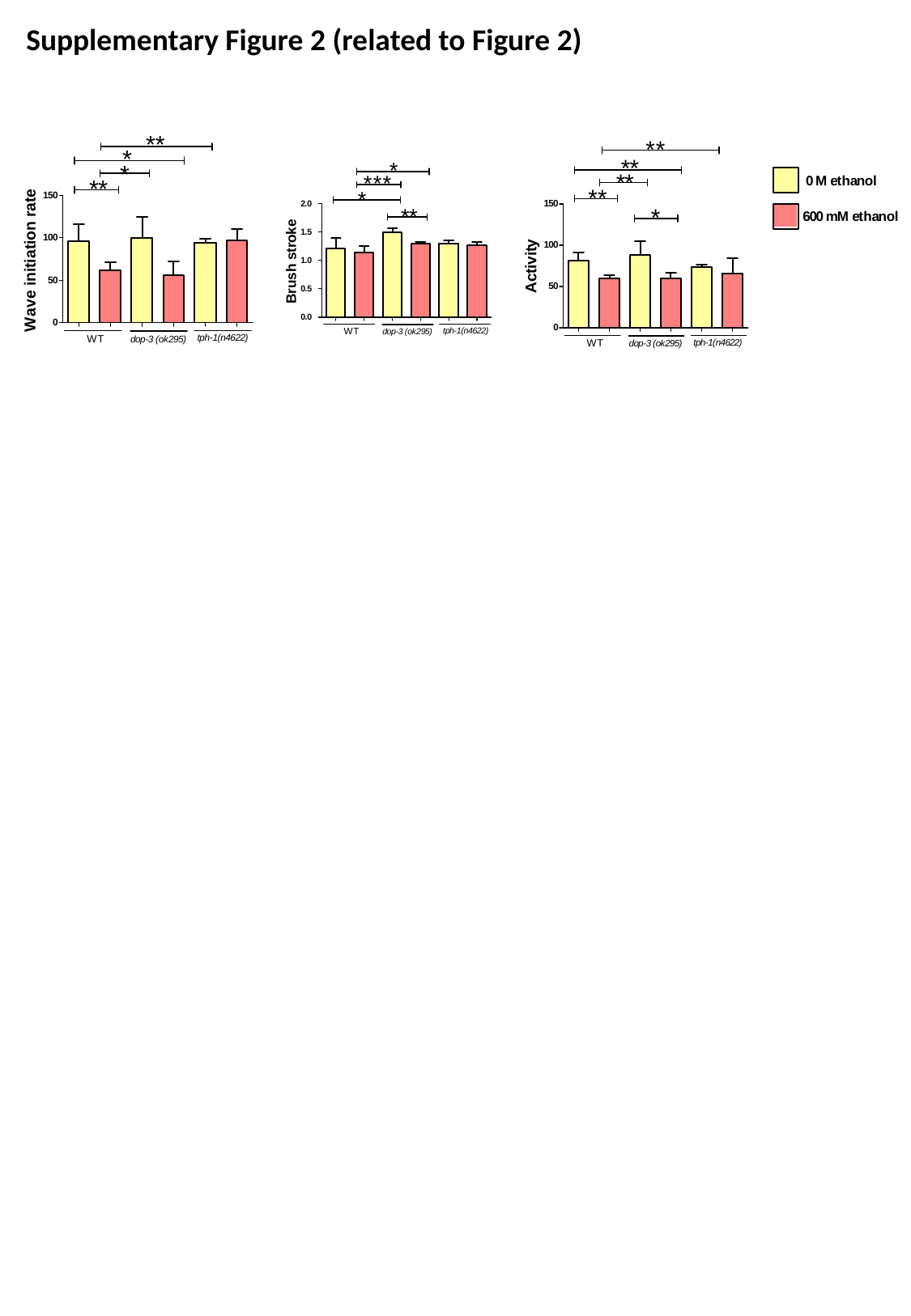

Supplementary Figure 2 (related to Figure 2)

Supplement: S2 Fig — Panels plot worm locomotion data of wild-type (N2 strain) animals, and dopamine (dop-3(ok295)) and serotonin (tph-1(n4622)) mutant strains from day 1 (D1) of adulthood, after 20 minute-treatment with 600 mM ethanol or no treatment, and 1 hour of post-exposure withdrawal period. Mean and standard deviation are depicted for each group. P-values were calculated by T-test corrected using the Benjamini–Hochberg false discovery rate (FDR) post hoc test (N = 5 independent experiments) *p < 0.05, **p < 0.01, ***p < 0.001. (PPTX) [file pone.0344966.s002.pptx]

## Slide 1
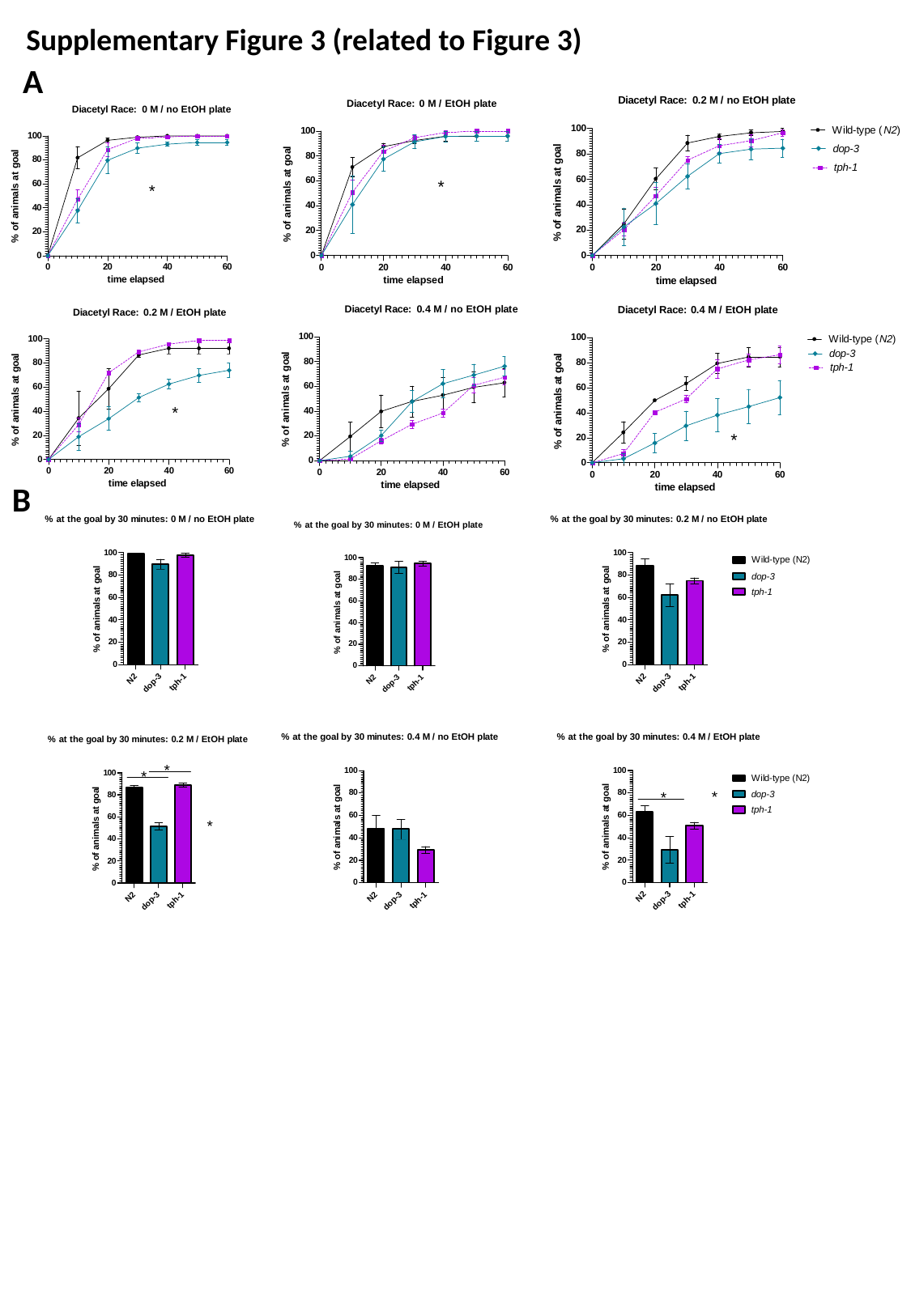

Supplementary Figure 3 (related to Figure 3)
A
B

Supplement: S3 Fig — Performance of wild-type (wild-type, N2 strain) animals, dopamine (dop-3(ok295)) and serotonin (tph-1(n4622)) mutant strains are depicted for each experimental condition (A) during the whole kinetic chemotaxis assay (“diacetyl race”) and (B) at 30 minutes. P-values were calculated by 1-way ANOVA for all conditions (indicated next to the curve/ bar graph) and by T-test for pairwise comparisons corrected using the Benjamini–Hochberg false discovery rate (FDR) post hoc test (at least N = 3 independent experiments): *p < 0.05. The results of 2-way ANOVA are shown in S2 Table. (PPTX) [file pone.0344966.s003.pptx]

## Slide 1
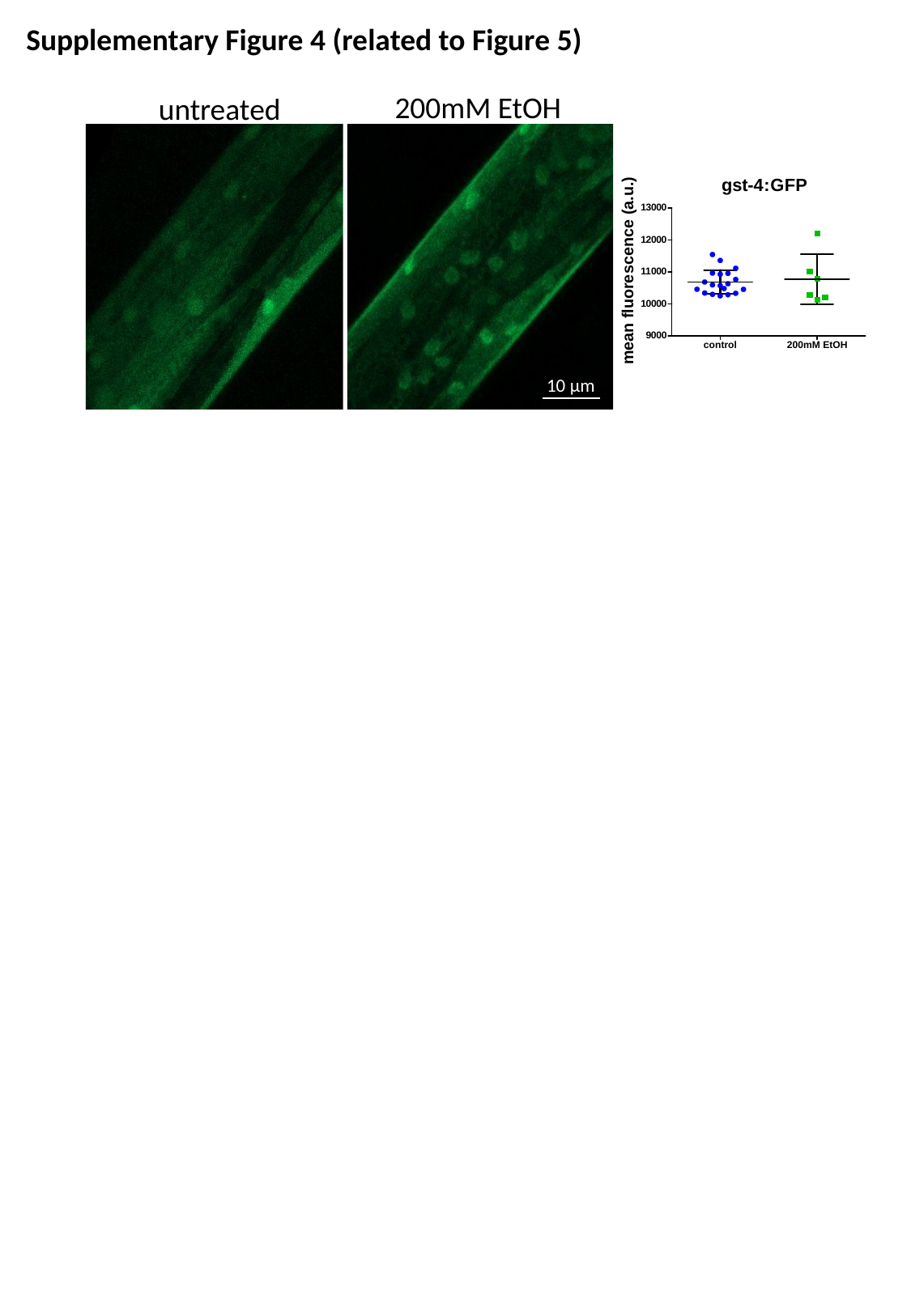

Supplementary Figure 4 (related to Figure 5)
200mM EtOH
untreated
10 μm

Supplement: S4 Fig — No statistically significant differences were detected using a T-test corrected using the Benjamini–Hochberg false discovery rate (FDR) post hoc test. (PPTX) [file pone.0344966.s004.pptx]
