# Supplementary material for: Specific genes of the dopaminergic (dop-3) and serotonergic (tph-1) pathways contribute to the effects of ethanol consumption in Caenorhabditis elegans
Source: PLoS One. 2026 Mar 23;21(3):e0344966. doi: 10.1371/journal.pone.0344966 (PMC13008063; doi:10.1371/journal.pone.0344966)
Supplement: S1 Table — (PPTX) [file pone.0344966.s005.pptx]

## Slide 1
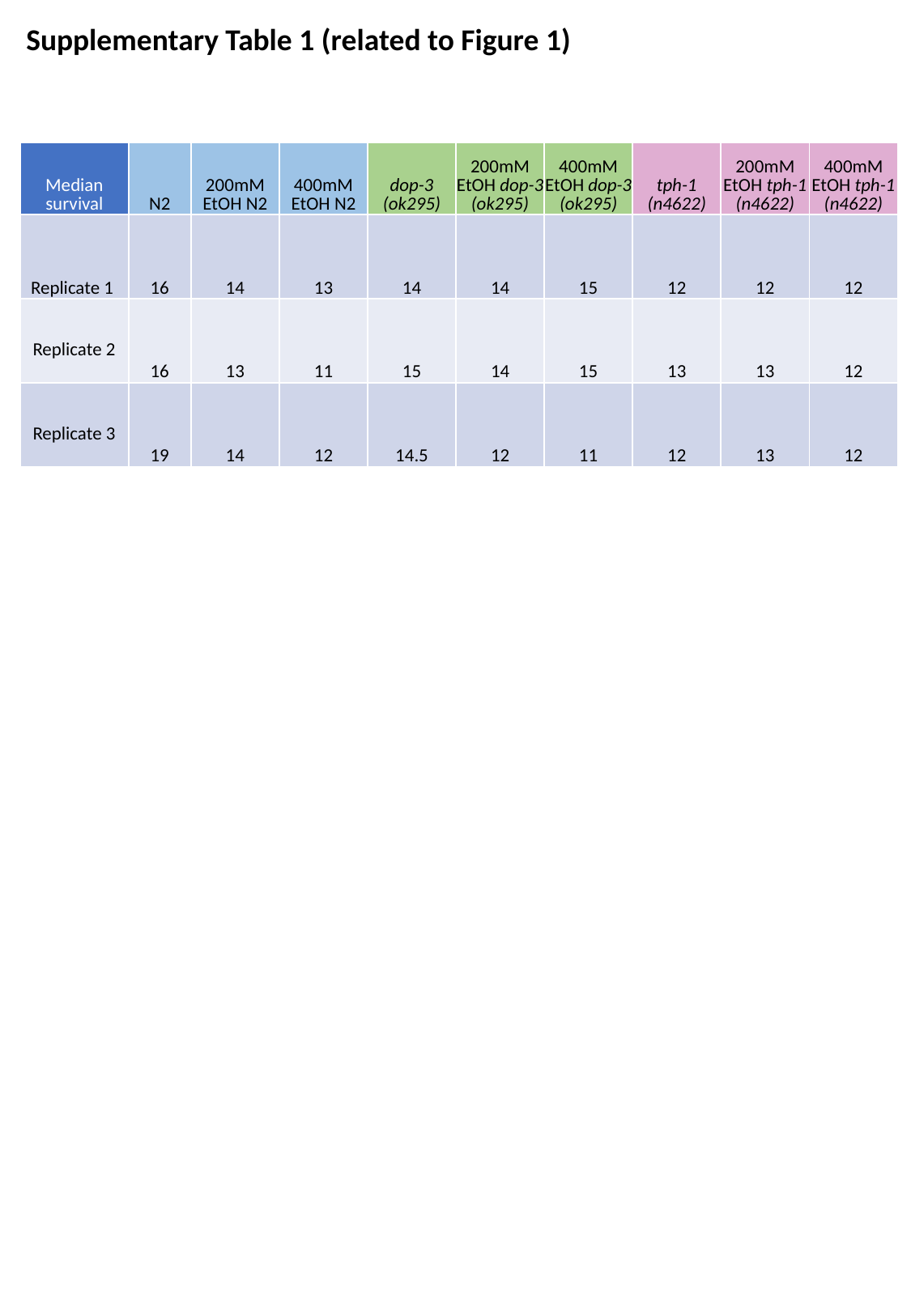

Supplementary Table 1 (related to Figure 1)
| Median survival | N2 | 200mM EtOH N2 | 400mM EtOH N2 | dop-3 (ok295) | 200mM EtOH dop-3 (ok295) | 400mM EtOH dop-3 (ok295) | tph-1 (n4622) | 200mM EtOH tph-1 (n4622) | 400mM EtOH tph-1 (n4622) |
| --- | --- | --- | --- | --- | --- | --- | --- | --- | --- |
| Replicate 1 | 16 | 14 | 13 | 14 | 14 | 15 | 12 | 12 | 12 |
| Replicate 2 | 16 | 13 | 11 | 15 | 14 | 15 | 13 | 13 | 12 |
| Replicate 3 | 19 | 14 | 12 | 14.5 | 12 | 11 | 12 | 13 | 12 |
