# Supplementary material for: Specific genes of the dopaminergic (dop-3) and serotonergic (tph-1) pathways contribute to the effects of ethanol consumption in Caenorhabditis elegans
Source: PLoS One. 2026 Mar 23;21(3):e0344966. doi: 10.1371/journal.pone.0344966 (PMC13008063; doi:10.1371/journal.pone.0344966)
Supplement: S2 Table — (DOCX) [file pone.0344966.s006.docx]

2-way ANOVA

| Figure 3B: Wild-type (*N2*) Diacetyl Race | | | | | |
| --- | --- | --- | --- | --- | --- |
| **Source of Variation** | **Df** | **Sum-of-squares** | **Mean square** | **F** | **P value** |
| Interaction | 30 | 10520 | 350.6 | 1.899 | 0.0117 |
| Dose | 5 | 24140 | 4827 | 26.15 | P<0.0001 |
| Time | 6 | 116400 | 19390 | 105 | P<0.0001 |
| Residual | 84 | 15510 | 184.6 |  |  |
|  |  |  |  |  |  |
| Figure 3C: Dopamine receptor null mutant (*dop-3*) Diacetyl Race | | | | | |
| **Source of Variation** | **Df** | **Sum-of-squares** | **Mean square** | **F** | **P value** |
| Interaction | 30 | 11640 | 387.9 | 1.664 | 0.0278 |
| Dose | 5 | 42720 | 8544 | 36.65 | P<0.0001 |
| Time | 6 | 134400 | 22390 | 96.05 | P<0.0001 |
| Residual | 126 | 29380 | 233.1 |  |  |
|  |  |  |  |  |  |
| Figure 3D: Serotonin null mutant (*tph-1*) Diacetyl Race | | | | | |
| **Source of Variation** | **Df** | **Sum-of-squares** | **Mean square** | **F** | **P value** |
| Interaction | 30 | 10510 | 350.2 | 7.464 | P<0.0001 |
| Dose | 5 | 33050 | 6610 | 140.9 | P<0.0001 |
| Time | 6 | 130300 | 21710 | 462.6 | P<0.0001 |
| Residual | 84 | 3942 | 46.92 |  |  |

| Supplementary Figure 3: Diacetyl Race: 0 M / no EtOH plate | | | | | |
| --- | --- | --- | --- | --- | --- |
| **Source of Variation** | **Df** | **Sum-of-squares** | **Mean square** | **F** | **P value** |
| Interaction | 12 | 1939 | 161.5 | 4.206 | 0.0002 |
| Genotype | 2 | 1065 | 532.7 | 13.87 | P<0.0001 |
| Time | 6 | 73580 | 12260 | 319.3 | P<0.0001 |
| Residual | 42 | 1613 | 38.41 |  |  |
|  |  |  |  |  |  |
| Supplementary Figure 3: Diacetyl Race: 0 M / EtOH plate | | | | | |
| **Source of Variation** | **Df** | **Sum-of-squares** | **Mean square** | **F** | **P value** |
| Interaction | 12 | 733 | 61.08 | 0.5003 | 0.9026 |
| Genotype | 2 | 146.2 | 73.12 | 0.599 | 0.554 |
| Time | 6 | 69340 | 11560 | 94.67 | P<0.0001 |
| Residual | 42 | 5127 | 122.1 |  |  |
|  |  |  |  |  |  |
| Supplementary Figure 3: Diacetyl Race: 0.2 M / no EtOH plate | | | | | |
| **Source of Variation** | **Df** | **Sum-of-squares** | **Mean square** | **F** | **P value** |
| Interaction | 12 | 754.3 | 62.86 | 0.4502 | 0.932 |
| Genotype | 2 | 613.6 | 306.8 | 2.198 | 0.1237 |
| Time | 6 | 74250 | 12380 | 88.65 | P<0.0001 |
| Residual | 42 | 5864 | 139.6 |  |  |
|  |  |  |  |  |  |
| Supplementary Figure 3: Diacetyl Race: 0.2 M / EtOH plate | | | | | |
| **Source of Variation** | **Df** | **Sum-of-squares** | **Mean square** | **F** | **P value** |
| Interaction | 12 | 2557 | 213.1 | 1.264 | 0.2753 |
| Genotype | 2 | 7040 | 3520 | 20.87 | P<0.0001 |
| Time | 6 | 60660 | 10110 | 59.95 | P<0.0001 |
| Residual | 42 | 7083 | 168.6 |  |  |
|  |  |  |  |  |  |
| Supplementary Figure 3: Diacetyl Race: 0.4 M / no EtOH plate | | | | | |
| **Source of Variation** | **Df** | **Sum-of-squares** | **Mean square** | **F** | **P value** |
| Interaction | 12 | 2817 | 234.7 | 1.06 | 0.416 |
| Genotype | 2 | 1370 | 685 | 3.094 | 0.0558 |
| Time | 6 | 40120 | 6687 | 30.21 | P<0.0001 |
| Residual | 42 | 9298 | 221.4 |  |  |
|  |  |  |  |  |  |
| Supplementary Figure 3: Diacetyl Race: 0.4 M / EtOH plate | | | | | |
| **Source of Variation** | **Df** | **Sum-of-squares** | **Mean square** | **F** | **P value** |
| Interaction | 12 | 3384 | 282 | 1.369 | 0.2189 |
| Genotype | 2 | 10200 | 5099 | 24.75 | P<0.0001 |
| Time | 6 | 44030 | 7338 | 35.62 | P<0.0001 |
| Residual | 42 | 8653 | 206 |  |  |
